# Pretreatment tissue cytokine signatures associate with paradoxical eczema in psoriasis under IL-17A blockade

**DOI:** 10.3389/fcell.2026.1893307

**Published:** 2026-08-13

**Authors:** Jingwen Wu, Youcong Wang, Xia Li, Li Zhang

**Affiliations:** Department of Dermatology, Rui Jin Hospital, Shanghai Jiao Tong University School of Medicine, Shanghai, China

**Keywords:** cytokine profiling, eczematous eruption, interleukin-17A inhibitor, psoriasis, skin homogenate

Inhibitors of interleukin-17A (IL-17A) afford strong clinical benefit in psoriasis; nevertheless, a minority of recipients paradoxically acquire eczematous lesions while on therapy, a phenomenon termed biologic-induced immune switching. While multi-omics approaches have mapped global inflammatory networks, a practical tissue-based assay to capture local immune states before therapy remains lacking (Hawkes et al., 2017). Because measurements obtained from serum frequently misrepresent the immune state confined to a lesion (Bai et al., 2018), assays that better capture the microenvironment of the plaque are urgently required. Our earlier work applied tissue homogenization to interrogate intralesional cytokines directly and showed that pronounced IL-21 elevation was far easier to detect in homogenates than in circulating blood (Zhang et al., 2026). Such observations imply that homogenate-based assessment holds particular promise for resolving the immunological character of cutaneous lesions. Here, we test whether lesional immune profiling, which better reflects tissue-specific inflammation than blood-based markers, can identify psoriatic patients predisposed to eczematous reactions under IL-17A blockade—an approach that complements existing clinical descriptions of biologic-induced immune switching. We explicitly separate diagnostic discrimination from prognostic exploration, both treated as hypothesis-generating.

This study was conducted at the Department of Dermatology, Ruijin Hospital, Shanghai Jiao Tong University School of Medicine. The first part of the study included serum samples from 17 patients with psoriasis vulgaris (mean age 60.1 years, 76% male, PASI 17.1) and 12 patients with atopic dermatitis (AD) (63.6 years, 58% male), as well as lesional skin tissue samples from 10 patients with psoriasis and 10 patients with AD. Ten healthy controls were included in PCA as reference controls but were not part of formal statistical comparisons. The second part retrospectively included psoriasis patients treated with IL-17A inhibitors. Psoriasis vulgaris patients were followed up for over 2 years and categorized into groups based on whether they subsequently developed an eczematous eruption (EE group) or did not experience this adverse event (PSV group). Serum samples were collected from 15 patients who developed eczematous eruptions (59.9 years, 60% male, PASI 14.1 and 17 who did not (60.1 years, 76% male, PASI 17.1, while lesional skin tissue samples were obtained from 10 patients in the eczematous eruption group and 10 in the non-eczematous eruption group.

Specimens reflecting the untreated state were secured ahead of biologic dosing. Using a 2-mm punch, full-thickness tissue was sampled from the center of a representative psoriatic plaque. Each 2-mm punch was homogenized in 300 µL RIPA lysis buffer (with protease/phosphatase inhibitors) using bead-based disruption (TissueLyser II, 3 cycles of 30 s at 4 °C), followed by centrifugation at 14,000 × g for 15 min at 4 °C. For serum, blood was collected in serum separator tubes, allowed to clot for 30 min at room temperature, and centrifuged at 1,500 × g for 15 min at 4 °C; hemolyzed samples were excluded. Serum and homogenate samples were measured in the same assay run to avoid batch effects. Total protein was quantified by BCA assay, and cytokine levels were normalized to total protein (pg/mg). All assays were run in duplicate with an inter-assay CV <10%. Then, cytokine amounts were determined on a bead-based multiplex flow-cytometry platform. PCA was performed on log10-transformed and z-score standardized values for all 12 analytes using the same scaling parameters for serum and homogenate datasets.

**FIGURE 1:**
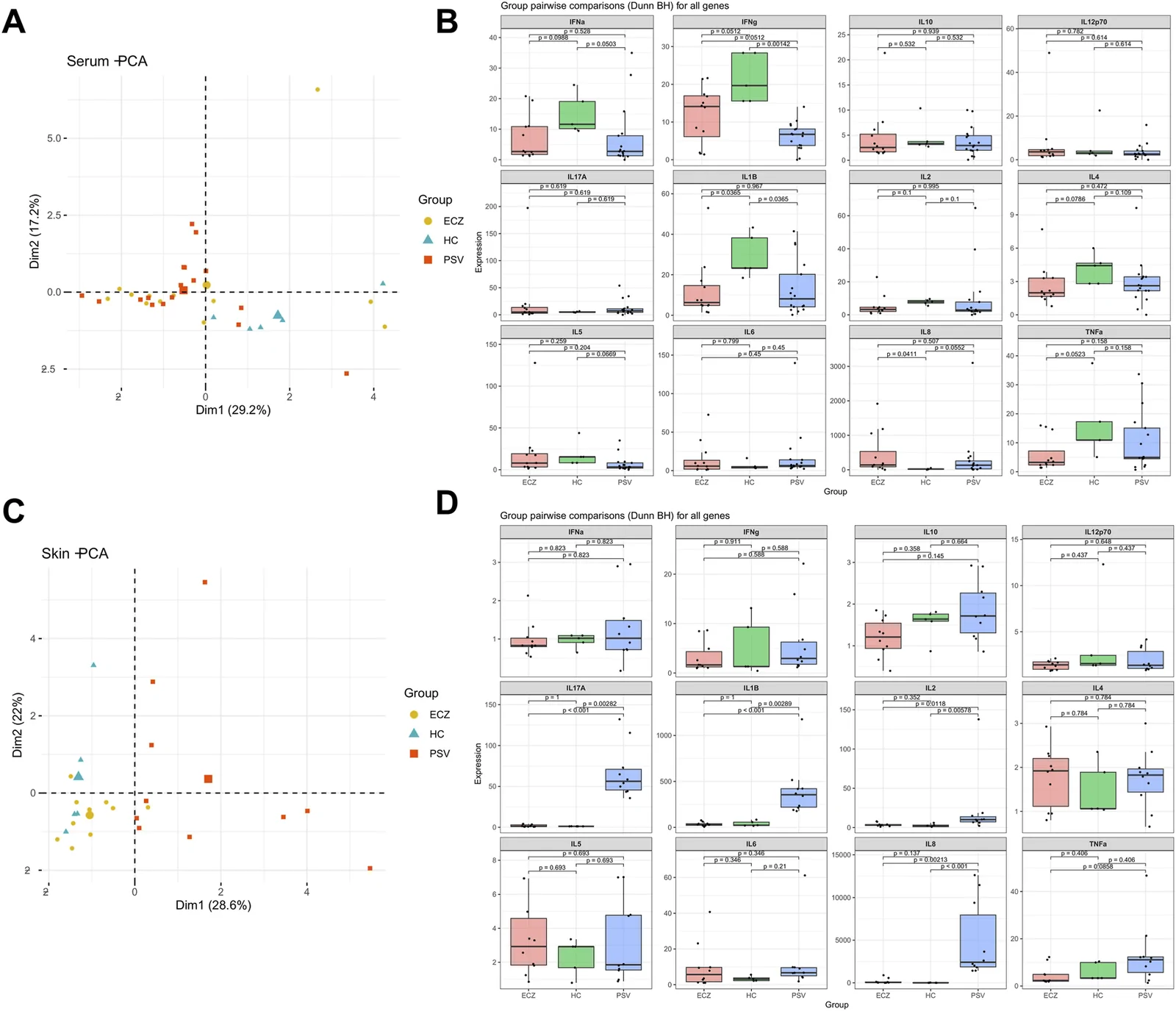
Serum versus skin cytokine profiles contrasted across psoriasis and atopic dermatitis. **(A)** PCA of serum cytokines in PSV (n = 17), AD (n = 12; labeled as ESZ), and HC (n = 10; reference only). **(B)** Boxplots of serum cytokine levels. **(C)** PCA of skin homogenate cytokines in PSV (n = 10), AD (n = 10), and HC (n = 10; reference only). **(D)** Boxplots of skin homogenate cytokines. Skin values normalized to total protein (pg/mg). Data are medians with IQRs; P from Mann–Whitney U tests; nominal significance indicated. PCA used log_10_-transformed, z-score-standardized values. PSV, psoriasis vulgaris; HC, healthy control (reference only); ESZ in figure denotes AD.

To begin, we asked whether interrogating the lesion adds discriminatory value over markers of systemic inflammation, contrasting Th2-skewed AD with Th17-skewed psoriasis. Principal component analysis (PCA) of serum cytokines showed substantial overlap between the two conditions (Figure 1A). Homogenate-derived cytokine patterns, by comparison, sharply separated the contrasting inflammatory states (Figure 1B). Indeed, while circulating mediators were statistically indistinguishable (Figure 1C), psoriatic plaques showed pronounced enrichment of IL-6, IL-8, IL-1β and IL-17A (Figure 1D). Collectively, these results reinforce the notion that homogenates capture immune fingerprints unique to each disease far better than blood.

**FIGURE 2:**
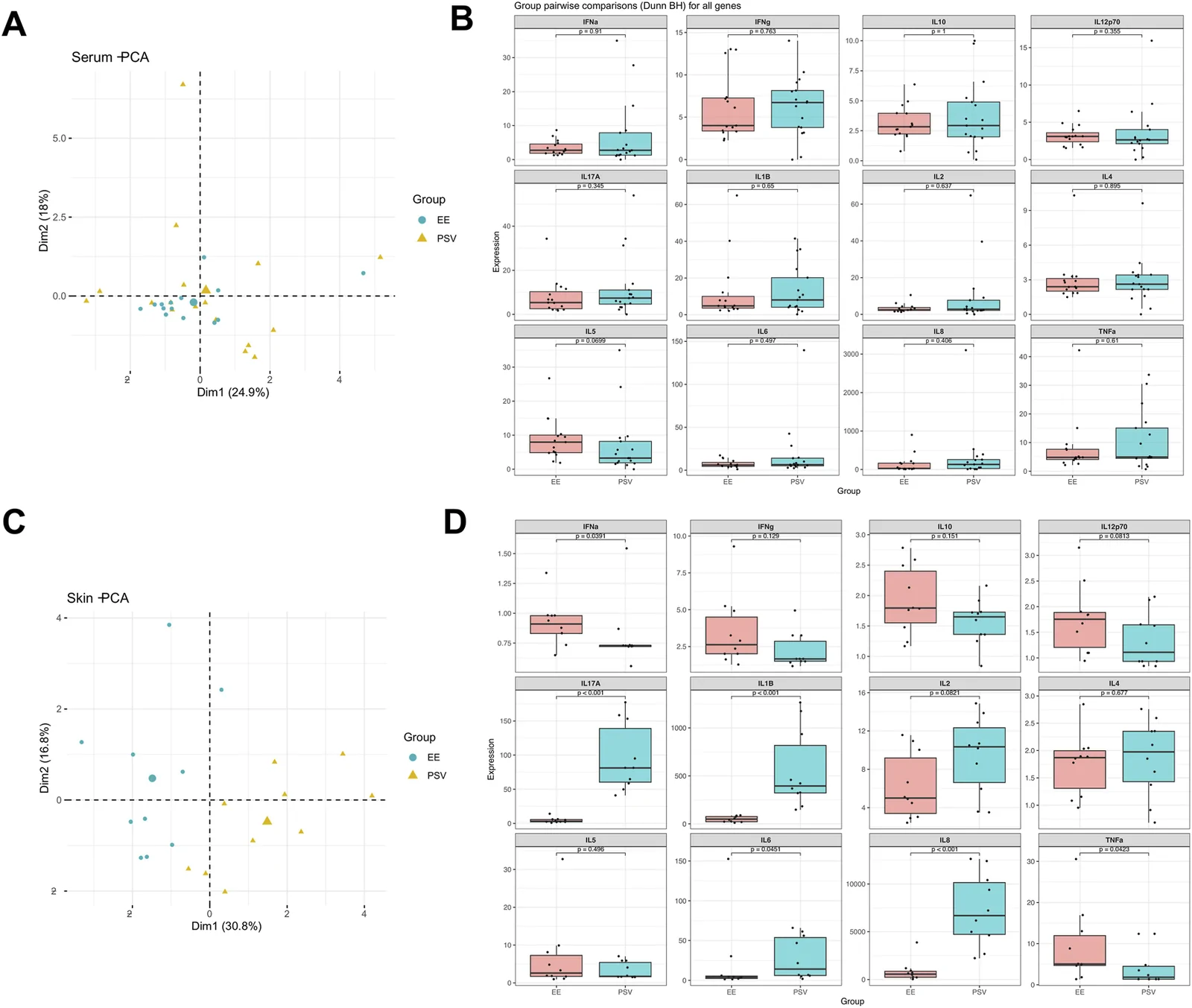
Pretreatment skin immune signatures associate with eczematous eruptions under IL-17A blockade. **(A)** PCA of baseline serum cytokines in patients who later developed eczematous eruptions (EE, n = 15) and those who did not (PSV, n = 17). **(B)** Boxplots of baseline serum cytokine levels for the EE and PSV groups. **(C)** PCA of baseline skin homogenate cytokines in EE (n = 10) and PSV (n = 10). **(D)** Boxplots showing skin homogenate cytokine levels in EE versus PSV. All skin cytokine values are normalized to total protein (pg/mg protein). Data are shown as medians with interquartile ranges; P values are from Mann–Whitney U tests with Benjamini–Hochberg FDR correction; nominal significance is indicated. PCA was performed on log_10_-transformed and z-score-standardized values.

Extending this finding, we examined how useful lesional cytokine measurement might be for associated with subsequent eczematous eruption. Baseline lesional skin and serum samples were collected prior to the first biologic administration (median 6 days; range 1–21 days); serum samples were available for a larger proportion of patients than lesional skin samples. Eczematous eruptions were diagnosed by two independent dermatologists (blinded to cytokine data) based on compatible clinical morphology and histopathological features (spongiosis, lymphocytic infiltration, and parakeratosis), after excluding allergic contact dermatitis, drug eruptions, and cutaneous infections through appropriate patch testing and microbiological evaluation. Serum signatures discriminated poorly between psoriatic patients destined to develop eczematous dermatitis while the remainder were spared (Figures 2A,B). Pretreatment cutaneous patterns, conversely, diverged substantially across the two cohorts (Figures 2C,D). In particular, individuals who subsequently progressed to eczematous dermatitis began with notably depressed IL-17A (18.3 vs. 72.6) together with allied pro-inflammatory mediators such as IL-6 (12.4 vs. 45.7), IL-8 (21.6 vs. 68.4) and IL-1β (9.8 vs. 31.2), whereas IFN-α (28.6 vs. 12.3) and TNF-α (41.2 vs. 22.7) were relatively elevated in lesional skin compared with the non-eczema group. After Benjamini–Hochberg FDR correction for multiple comparisons, IL-8 (q = 0.06) and IFN-α (q = 0.09) showed marginal between-group differences, whereas IL-4, IL-5, and IL-10 did not reach the adjusted threshold (q > 0.10). Effect sizes (Cliff’s delta) for IL-17A, IL-6, IL-8, IL-1β, IFN-α, and TNF-α ranged from 0.72 to 0.89, indicating large-magnitude differences.

Biologic-induced immune switching, wherein IL-17A blockade unmasks alternative inflammatory axes (e.g., IFN-α/TNF-α), has been increasingly recognized in psoriasis. Our targeted tissue-based findings align with this concept and complement recent multi-omics and computational frameworks (Singaravel and Moovendhan, 2025; Vijayapoopathi et al., 2023), offering a localized, interpretable readout that bridges discovery and clinical applicability. Taken together, these data imply that individuals susceptible to eczematous dermatitis under IL-17A blockade may start from a characteristic cutaneous immune state in which Th17-driven activity is comparatively muted. Adding further IL-17A suppression onto such a background might theoretically disrupt local immune balance, potentially favoring competing inflammatory programs. The elevated IFN-α may reflect pre-existing type I interferon activity, potentially from plasmacytoid dendritic cell activation, which has been implicated in both psoriasis heterogeneity and early eczematous responses. However, this mechanistic interpretation remains speculative and requires functional validation.

Beyond discriminative potential, the 2-mm-punch homogenate assay used here carries clear practical merits for the clinic. To begin with, whereas single-cell sequencing demands costly handling and freshly processed tissue within tight time windows, preparing homogenate specimens follows a thoroughly standardized routine. Proteins recovered in this way remain markedly stable, which eases both storage and shipment. In contrast to tape-stripping methods, our approach offers quantitative readouts of tissue mediators (Chen et al., 2025; He et al., 2021). Our approach is distinguished by its ability to yield exact, quantitative readouts of inflammatory mediators in the tissue (normalized to pg/mg), furnishing dependable evidence for steering treatment. Lastly, a 2-mm wound generally needs no sutures, closes quickly with negligible scarring, and costs little, all of which markedly improve patient acceptance. Nevertheless, patient compliance, procedure time, and cost-effectiveness relative to non-invasive alternatives (e.g., tape stripping) remain practical barriers that may limit routine adoption to selected high-risk scenarios.

This work has several methodological constraints. First, despite applying Benjamini–Hochberg FDR correction, the small sample size limits statistical power, and no multivariate modeling (e.g., LASSO or logistic regression) was applied to account for cytokine interactions or confounding; thus, the reported differences should be interpreted as exploratory and hypothesis-generating rather than confirmatory. Second, the small sample size and retrospective design further limit statistical robustness. Third, the panel omitted several eczema-relevant mediators, such as IL-13. Confirmation will require larger studies of prospective design. While multi-omics approaches provide broad molecular landscapes, their complexity and cost limit routine clinical application. In contrast, targeted cytokine profiling from tissue homogenates offers a standardized, cost-effective, and clinically feasible alternative that captures local inflammatory signatures with high fidelity.

To conclude, quantifying cytokines in tissue homogenates may better reflect local inflammation than serum and could help characterize immune shifts associated with eczematous risk. These findings position homogenate analysis as a candidate stratification tool warranting prospective validation rather than a ready-to-use clinical decision aid. External validation in an independent cohort is required before clinical translation.
